# Migraine and hemorrhagic stroke: data from general practice

**DOI:** 10.1186/1129-2377-16-8

**Published:** 2015-01-21

**Authors:** Raffaele Ornello, Francesca Pistoia, Diana Degan, Antonio Carolei, Simona Sacco

**Affiliations:** Institute of Neurology, Department of Applied Clinical Sciences and Biotechnology, University of L’Aquila, 67100 L’Aquila, Italy

**Keywords:** Migraine, Stroke, Intracerebral hemorrhage

Migraine is one of the most disabling headache disorders and the seventh most disabling disease worldwide [1]. Several epidemiological studies have investigated the association between migraine and vascular disease [2]. Two meta-analyses found a clear increase in the risk of ischemic stroke in subjects with migraine, mostly migraine with aura (MA), as compared with non-migraineurs [3, 4]. Another meta-analysis found an increased risk of hemorrhagic stroke (HS) in subjects with migraine [5], although potential sources of heterogeneity can be found among the studies included in that analysis. Most studies did not distinguish between intracerebral hemorrhage (ICH) and subarachnoid hemorrhage (SAH) when assessing the outcomes and some of them did not report the outcomes separately for MA and migraine without aura (MO). In addition, in the available studies little consideration was given to migraine activity and duration.

The case–control study published in *The Journal of Headache and Pain* by Gaist and colleagues [6] adds important data gathered from general practice to the link between migraine and vascular diseases. In that study, data from 1,797 subjects with ICH and 1,340 subjects with SAH from a large epidemiological dataset, The Health Improvement Network (THIN), were reviewed and frequency-matched with control subjects for sex, age (±1 year), and calendar year of diagnosis. After adjustment for sex, age, calendar year, smoking, alcohol, body mass index, hypertension, previous cerebrovascular disease, oral contraceptive use, and health services utilization, the authors did not find an increased risk of overall HS or of ICH or SAH in subjects with migraine compared with non-migraineurs. Analysis according to migraine type showed that neither MA nor MO were associated with an increased risk of HS, ICH or SAH [6]. Only subjects with a long history (≥20 years) of migraine had an increased risk of ICH compared to control subjects, and even they did not show an increased risk of SAH [6]. The results of this study conflicted somewhat with previous studies [5, 7–10] which indicated that the overall increase in the risk of HS associated with migraine was mostly driven by ICH rather than SAH and by MA rather than MO.

To understand the discrepancies we should consider the strengths and limitations of that study. The enrolment of a large number of patients with HS in a general practice setting confers an advantage to this new study. In addition, the authors performed separate analyses for ICH and SAH, well known clinical entities with different pathogenesis. Most importantly, this is, to our knowledge, the first case–control study that includes migraine duration in the statistical analyses suggesting a correlation between time from migraine diagnosis and risk of vascular events. This is in line with the Women’s Health Study which found evidence of the increased risk of HS in migraineurs only after the 13.6 years of follow-up [10]. Likewise, when considering the risk of cardiac ischemic disease in migraineurs, the association between migraine and coronary heart disease was evident only after not less than ten years of follow-up [11–13]. This finding contrasts with the risk of ischemic stroke, which is increased even in studies with shorter follow-up [14]. These same data suggest that the vascular risk associated with migraine increases over time, with variable effects on different vascular diseases. However, the study by Gaist et al. has some limitations that suggest caution in considering their findings as conclusive. Migraine diagnosis was ascertained retrospectively and was not confirmed by headache experts, thus potentially introducing a bias. Criteria for migraine diagnosis were not standardized and not comparable to those of the International Classification of Headache Disorders [15]. Pitfalls in diagnosis might have been even greater when addressing migraine subtypes since some auras may have been missed or misdiagnosed. Besides, authors did not describe how they managed cases with incomplete records or with headaches attributable to probable migraine. Moreover, migraine duration was measured as the time from recorded diagnosis to the follow-up event date; for the same reason authors were not able to assess migraine activity at the time of the vascular event. Previous findings suggest that the correlation between MA and HS was significant only in subjects with active migraine, but not in subjects with past history of migraine [10].

The association between migraine and HS is still unanswered, because of several issues. ICH and SAH are rarer than ischemic stroke in high-income countries [16], and very large populations are needed to assess the association between HS and migraine. Electronic datasets, offering the opportunity to compute huge networks of data, could help in overcoming that problem. However, data on diagnoses are often collected for purposes other than research or medical surveillance [17] and this potentially hinders their accuracy. Therefore, the design of new epidemiological studies should include external validation of exposures and outcomes. Indeed, there is a need for further large, prospective, population-based cohort studies assessing the relationships between migraine subtypes (MA, MO), migraine status (active, inactive) and migraine duration and the risk of ICH and SAH. Well-designed studies should also help explaining the role of potential confounders of the association between migraine and HS, such as the chronic assumption of non-steroidal anti-inflammatory drugs (NSAIDs) which have an anti-platelet and hypertensive action and have been associated, by some studies, with stroke [18]; among the available studies, only the one performed within the Women’s Health Study [10] ran an analysis with adjustment for time varying frequency of NSAIDs intake and randomized aspirin assignment; however, that study found no change in the associations after that adjustment. Finally, it should be noted that migraine and HS have some vascular comorbidities [19–21], particularly hypertension, which is one of the most important risk factors for HS [22]; however, the great majority of the studies investigating the association between migraine and HS adjusted their statistical models for hypertension. Future studies should be planned to verify the presence of any vascular vulnerability in migraine in order to explain the increased vascular risk of migraineurs and to develop new therapies acting on both migraine and the associated vascular risk.

## References

[1] Steiner TJ, Stovner LJ, Birbeck GL (2013). Migraine: the seventh disabler. Headache.

[2] Sacco S, Ricci S, Carolei A (2012). Migraine and vascular diseases: a review of the evidence and potential implications for management. Cephalalgia.

[3] Schürks M, Rist PM, Bigal ME, Buring JE, Lipton RB, Kurth T (2009). Migraine and cardiovascular disease: systematic review and meta-analysis. BMJ.

[4] Spector JT, Kahn SR, Jones MR, Jayakumar M, Dalal D, Nazarian S (2010). Migraine headache and ischemic stroke risk: an updated meta-analysis. Am J Med.

[5] Sacco S, Ornello R, Ripa P, Pistoia F, Carolei A (2013). Migraine and hemorrhagic stroke: a meta-analysis. Stroke.

[6] Gaist D, González-Pérez A, Ashina M, García Rodríguez LA (2014). Migraine and risk of hemorrhagic stroke: a study based on data from general practice. J Headache Pain.

[7] Carter KN, Anderson N, Jamrozik K, Hankey G, Anderson CS, Australasian Co-operative Research on Subarachnoid Haemorrhage Study (ACROSS) Group (2005). Migraine and risk of subarachnoid haemorrhage: a population-based case–control study. J Clin Neurosci.

[8] Chang CL, Donaghy M, Poulter N (1999). Migraine and stroke in young women: case–control study. The World Health Organisation Collaborative Study of Cardiovascular Disease and Steroid Hormone Contraception. BMJ.

[9] Kuo CY, Yen MF, Chen LS, Fann CY, Chiu YH, Chen HH, Pan SL (2013). Increased risk of hemorrhagic stroke in patients with migraine: a population-based cohort study. PLoS ONE.

[10] Kurth T, Kase CS, Schürks M, Tzourio C, Buring JE (2010). Migraine and risk of haemorrhagic stroke in women: prospective cohort study. BMJ.

[11] Cook NR, Benseñor IM, Lotufo PA, Lee IM, Skerrett PJ, Chown MJ, Ajani UA, Manson JE, Buring JE (2002). Migraine and coronary heart disease in women and men. Headache.

[12] Kurth T, Gaziano JM, Cook NR, Logroscino G, Diener HC, Buring JE (2006). Migraine and risk of cardiovascular disease in women. JAMA.

[13] Kurth T, Gaziano JM, Cook NR, Bubes V, Logroscino G, Diener HC, Buring JE (2007). Migraine and risk of cardiovascular disease in men. Arch Intern Med.

[14] Buring JE, Hebert P, Romero J, Kittross A, Cook N, Manson J, Peto R, Hennekens C (1995). Migraine and subsequent risk of stroke in the Physicians’ Health Study. Arch Neurol.

[15] Headache Classification Subcommittee of the International Headache Society (2004). The International Classification of Headache Disorders: 2nd edition. Cephalalgia.

[16] Sacco S, Stracci F, Cerone D, Ricci S, Carolei A (2011). Epidemiology of stroke in Italy. Int J Stroke.

[17] Sacco S, Pistoia F, Carolei A (2013). Stroke tracked by administrative coding data: is it fair?. Stroke.

[18] Park K, Bavry AA (2014). Risk of stroke associated with nonsteroidal anti-inflammatory drugs. Vasc Health Risk Manag.

[19] Tana C, Tafuri E, Tana M, Martelletti P, Negro A, Affaitati G, Fabrizio A, Costantini R, Mezzetti A, Giamberardino MA (2013). New insights into the cardiovascular risk of migraine and the role of white matter hyperintensities: is gold all that glitters?. J Headache Pain.

[20] Sacco S, Olivieri L, Bastianello S, Carolei A (2006). Comorbid neuropathologies in migraine. J Headache Pain.

[21] Sacco S, Cerone D, Carolei A (2008). Comorbid neuropathologies in migraine: an update on cerebrovascular and cardiovascular aspects. J Headache Pain.

[22] Ariesen MJ, Claus SP, Rinkel GJ, Algra A (2003). Risk factors for intracerebral hemorrhage in the general population: a systematic review. Stroke.

